# Socioeconomic and Psychosocial Adversity in Midlife and Depressive Symptoms Post Retirement: A 21-year Follow-up of the Whitehall II Study

**DOI:** 10.1016/j.jagp.2014.04.001

**Published:** 2015-01

**Authors:** Marianna Virtanen, Jane E. Ferrie, G. David Batty, Marko Elovainio, Markus Jokela, Jussi Vahtera, Archana Singh-Manoux, Mika Kivimäki

**Affiliations:** a Finnish Institute of Occupational Health, Helsinki, Finland; b Department of Epidemiology and Public Health, University College London, United Kingdom; c Centre for Cognitive Ageing & Cognitive Epidemiology, University of Edinburgh, United Kingdom; d School of Community and Social Medicine, University of Bristol, Bristol, United Kingdom; e National Institute for Health and Welfare, Helsinki, Finland; f Institute of Behavioral Sciences, University of Helsinki, Helsinki, Finland; g Department of Public Health, University of Turku and Turku University Hospital, Finland; h Inserm U1018, Centre for Research in Epidemiology and Population Health, Villejuif, France

**Keywords:** Depression, elderly, inequalities, life course, mood disorders, old age, prospective, stress

## Abstract

**Objective:**

We examined whether socioeconomic and psychosocial adversity in midlife predicts post-retirement depressive symptoms.

**Design and Setting:**

A prospective cohort study of British civil servants who responded to a self-administered questionnaire in middle-age and at older ages, 21 years later.

**Participants:**

The study sample consisted of 3,939 Whitehall II Study participants (2,789 men, 1,150 women; mean age 67.6 years at follow-up) who were employed at baseline and retired at follow-up.

**Measurements:**

Midlife adversity was assessed by self-reported socioeconomic adversity (low occupational position; poor standard of living) and psychosocial adversity (high job strain; few close relationships). Symptoms of depression post-retirement were measured by the Center for Epidemiologic Studies Depression scale.

**Results:**

After adjustment for sociodemographic and health-related covariates at baseline and follow-up, there were strong associations between midlife adversities and post-retirement depressive symptoms: low occupational position (odds ratio [OR]: 1.70, 95% confidence interval [CI]: 1.15–2.51), poor standard of living (OR: 2.37, 95% CI: 1.66–3.39), high job strain (OR: 1.52, 95% CI: 1.09–2.14), and few close relationships (OR: 1.51, 95% CI: 1.12–2.03). The strength of the associations between socioeconomic, psychosocial, work-related, or non-work related exposures and depressive symptoms was similar.

**Conclusions:**

Robust associations from observational data suggest that several socioeconomic and psychosocial risk factors for symptoms of depression post-retirement can be detected already in midlife.

Symptoms of depression are common in old age; 8%–20% of elderly populations have reported depressive mood or depressive symptoms in epidemiological studies.^1^, ^2^, ^3^, ^4^ Depression impairs quality of life, increases health care costs, and is associated with premature mortality.^5^, ^6^, ^7^, ^8^, ^9^, ^10^, ^11^ Given the current size and projected growth in the older population globally, it is important to identify modifiable risk factors for depression in this group. Genetic factors, recurrent depressive episodes, physical and psychiatric comorbidity, and socioeconomic and psychosocial adversities, assessed in later life, have been shown to be associated with an elevated risk of depression.^2^, ^6^, ^12^, ^13^, ^14^

Childhood adversities, midlife negative life events, and marital stress have been shown to have long-term effects on depression in old age.^15^ There is also considerable evidence that exposure to socioeconomic^14^, ^16^ and psychosocial adversity (e.g., work stress) is associated with depression in midlife.^17^, ^18^ However, the extent to which exposures during employment—many of which are amenable to intervention—have predictive value beyond retirement has not been established. There is some evidence that social inequalities in physical health, indexed as self-rated general health,^19^ mental well-being,^20^ and mortality,^21^ may persist after retirement and that stress at work predicts poorer post-retirement mental and physical well-being.^22^ To the best of our knowledge there has been no study of post-retirement depressive symptoms as an outcome. Moreover, in any such investigation the contribution of poor physical health and health risk behaviors in old age should be taken into account as they are plausible sources of confounding or reverse causation given that depression, physical diseases, and health risk behaviors tend to cluster in the same individuals.

In this study, we used data from the Whitehall II cohort to examine whether midlife socioeconomic and psychosocial adversity is associated two decades later with symptoms of depression post-retirement, and whether any observed associations are attributable to midlife mental health and post-retirement sociodemographic factors, physical health, and health risk behaviors.

## Methods

### Participants and Study Design

The Whitehall II study is a prospective cohort study of British civil servants (government employees) established to identify social and environmental determinants of pathophysiological changes and disease.^23^ Ethical approval for the Whitehall II study was obtained from the University College London Medical School committee on the ethics of human research; all participants provided written informed consent. The target population was all London-based office staff, aged 35–55 years, working in 20 civil service departments on recruitment to the study in 1985–1988. With a response proportion of 73%, the cohort consisted of 10,308 employees. Since then, eight follow-up examinations have taken place approximately every 2 to 3 years.

**Figure 1:**
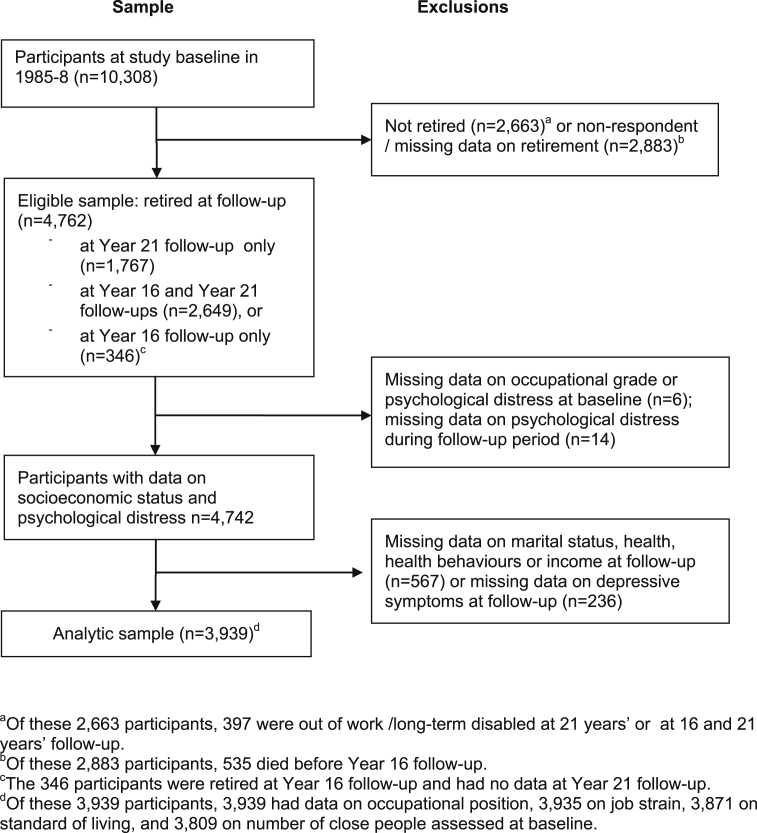
Sample selection procedure, the Whitehall II Study.

The Center for Epidemiologic Studies Depression (CES-D) scale,^24^ used as the outcome in the present study, was first introduced at the 2002–2004 examination and repeated in 2007–2009. For the analysis we selected participants who were (according to survey responses) retired due to old age either in 2007–2009 (N = 1,767, group 1) or both in 2002–2004 and 2007–2009 (N = 2,649, group 2), or were retired at 2002–2004 but non-respondents in 2007–2009 (N = 346, group 3) (Figure 1), resulting in an eligible sample of 4,762 men and women. Assessment of CES-D depressive symptoms was based on response in 2007–2009 in group 1, 2002–2004 and 2007–2009 in group 2 (presence of symptoms in either survey), and 2002–2004 in group 3. We excluded participants who had incomplete data on covariates, health-related variables, and missing data on CES-D at the follow-up and baseline occupational position or psychological distress at baseline and during the follow-up years (data based on surveys at 1989–1990, 1991–1993, 1997–1999, 2001, 2002–2004 and when psychological distress was assessed, depending on the length of follow-up of each participant), for a total of 823 participants. Thus the analytic sample comprised 3,939 participants; of these 2,789 were men, 1,150 were women, and the mean age was 45.9 (SD: 5.6) years at baseline and 67.6 (SD: 5.5) years at follow-up. The numbers in the analyses varied between 3,809 and 3,939 based on data available on baseline exposures (occupational position, job strain, standard of living, and number of close relationships).

### Exposures

Socioeconomic and psychosocial adversity measures were derived from the baseline survey in 1985–1988. In case of missing data, information was completed from surveys subsequent to baseline but prior to the 2002–2004 follow-up, requiring that the participant was still employed; the follow-up and length of follow-up was calculated accordingly. Socioeconomic adversity was assessed as occupational position and standard of living, and psychosocial adversity as job strain and number of close people.

#### Socioeconomic adversity

Occupational position at baseline was defined as low, average, and high.^25^ In the Whitehall II study it is a comprehensive marker of socioeconomic position and is related to salary, social status, and level of responsibility at work. The civil service identifies 12 non-industrial grades that, in order of increasing salary, comprise clerical assistant, clerical officer, executive officer, higher executive officer, senior executive officer, and seven unified grades. Other professional and technical staff are assigned to these grades on the basis of salary. For analysis, unified grades 1–6 were combined into one group and the bottom two clerical grades into another, producing six categories that in turn were collapsed to form the categories low, average, and high. Standard of living was ascertained using the following question: “All things considered, how satisfied or dissatisfied are you with your standard of living?” Response options ranged from 1 = very dissatisfied to 7 = very satisfied. A three-category variable was formulated to indicate poor (very/moderately dissatisfied), average (a little dissatisfied/no feelings either way/a little satisfied), and good (moderately/very satisfied).

#### Psychosocial adversity

The “job strain” model^26^ was used to describe work stress. For each participant, mean response scores were calculated for four job-demand items (i.e., questions about whether the participant had to work very hard, had excessive amounts of work, conflicting demands, or insufficient time) and 15 items on control over job-related decision-making and skill discretion (i.e., job control).^27^ High job demands were defined as a job-demand score greater than the median and low job control as a job control score lower than the median. We then classified the participants as low job strain (low demands and high job control), active job (high demands and high job control), passive job (low demands and low job control), and high job strain (high demands and low job control). Participants were asked to report the number of people they feel very close to (including people who have died and people they have not seen recently). The number of close people was then classified into three categories: 0–2, 3–6, and 7 or more.

### Outcome: Post-Retirement Depressive Symptoms

The 20-item CES-D scale^24^ was used to identify depressive symptomatology in retirement, approximately 16 years (2002–2004) and 21 years (2007–2009) after the assessment of the exposures. Participants were asked to score the frequency of occurrence of specific symptoms during the previous week on a four-point scale (0 = less than one day, 1 = 1–2 days, 2 = 3–4 days and 3 = 5–7 days). These are summed to yield a total score between 0 and 60, with participants scoring 16 or more defined as cases of CES-D depressive symptoms.^28^

### Covariates

The following potential confounders were assessed: sex; length of follow-up (years) between baseline and follow-up examinations, and from the 2002–2004 or 2007–2009 examinations; age; marital status (single, divorced/separated, widowed vs married/co-habiting); income (self-reported total family income divided by the number of income-earners; further classified as low, average, versus high income); self-reported longstanding illness (yes versus no): clinically verified coronary heart disease (yes versus no);^25^ smoking (yes versus no); alcohol use (heavy or frequent, moderate versus no); and physical functioning (low, average versus high) based on the Short Form 36 (SF-36) physical function score.^29^

In addition, we used the self-administered 30-item General Health Questionnaire (GHQ-30)^30^ to control for mental health status at baseline and during the follow-up years prior to the introduction of the CES-D in 2002–2004. The GHQ is widely used in large population-based surveys and trials and has been shown to have good predictive value in detecting clinical depression in the Whitehall II study population.^31^ Any participant with a score greater than 4 at baseline was defined as a GHQ-case.^31^ Participants who scored greater than 4 at least once at any subsequent phase prior to their assessment on the CES-D at follow-up were defined as GHQ-cases during the follow-up years.

### Statistical Analysis

All data on occupational position were from the baseline survey in 1985–1988. For job strain and baseline psychological distress (GHQ-cases), 99% of the data were from 1985–1988; for standard of living 75% were from 1985–1988, 23% from 1989–1990, and 2% from 1991–1993; and for the number of close persons, 74% were from 1985–1988, 25% from 1989–1990, and 2% from 1997–1999. The follow-up time for each participant for each exposure variable was calculated accordingly. For those participants whose status was retired at both the 2002–2004 and 2007–2009 examinations, follow-up time was calculated to 2002–2004 if CES-D depression was detected at that examination, otherwise it was set to 2007–2009. Mean follow-up time thus varied between 20.1 and 21.2 years depending on the exposure and outcome group.

We used logistic regression analysis to examine the adjusted associations between adversity in midlife and symptoms of depression post-retirement. Adjustments were carried out to control for baseline psychological distress and occupational position, length of follow-up, and potential post-retirement confounding factors (sociodemographic factors, health behaviors, physical health status) as well as the potential onset of psychological distress (as a proxy for depression) over the follow-up period. Trends were tested by adding the categorical exposure variables into the model as continuous. All analyses were carried out with the SAS 9.2 program package.

## Results

Table 1 presents the number of participants and number of cases by each exposure category, and associations between midlife adversities and post-retirement depressive symptoms. We identified 534 cases of depressive symptoms (13.6% of the participants). In the model adjusted for age, sex, and length of follow-up, all midlife socioeconomic and psychosocial adversities predicted symptoms of depression post-retirement; the corresponding odds ratios varied between 1.49 and 3.47. Of participants in low occupational positions and among those who reported a poor standard of living, 22.7% and 25.5%, respectively, had depressive symptoms 21 years later. Further adjustments for psychological distress at baseline and during the follow-up years, in addition to post-retirement health indicators, health behaviors, and income level, attenuated, but did not remove, the association of socioeconomic and psychosocial adversity with post-retirement depressive symptoms. With regard to occupational position, a larger reduction in the odds ratio (to 1.70) was observed after adjustment for post-retirement health related covariates and income. The odds ratio after leaving out post-retirement income from the final model was 1.95 (95% CI: 1.37–2.76 for low occupational position compared with high, logistic regression analysis [df = 2], p = 0.0002; data not shown), suggesting that the reduction of the estimate was in part explained by post-retirement low income, and in part by poor health and health risk behaviors. The association remained statistically significant at conventional levels after all adjustments.

**Table 1:** Odds Ratios (OR) and 95% Confidence Intervals (CI) Comparing Risk for Post-Retirement Depressive Symptoms by Exposure to Midlife Adversities

Midlife Adversity	Post-Retirement Depressive Symptoms														
	N	N of cases	%	OR^a^	95% CI^a^	Wald χ^2^ (df)	p	OR^b^	95% CI^b^	Wald χ^2^ (df)	p	OR^c^	95% CI^c^	Wald χ^2^ (df)	p
All	3,939	534	13.6												
Occupational position															
High	1,392	133	9.6	1.00				1.00				1.00			
Average	1,961	268	13.7	1.35	1.07–1.71	6.20 (1)	0.013	1.30	1.01–1.66	4.22 (1)	0.040	1.16	0.88–1.52	1.14 (1)	0.29
Low	586	133	22.7	2.13	1.55–2.93	21.72 (1)	<0.0001	2.33	1.67–3.24	24.85 (1)	<0.0001	1.70	1.15–2.51	7.03 (1)	0.008
p for trend						20.80 (1)	<0.0001			22.44 (1)	<0.0001			6.31 (1)	0.012
Standard of living	3,871	519	13.4												
Good	2,708	286	10.6	1.00				1.00				1.00			
Average	912	169	18.5	2.00	1.61–2.48	39.66 (1)	<0.0001	1.54	1.22–1.93	13.61 (1)	0.0002	1.50	1.19–1.89	11.61 (1)	0.0007
Poor	251	64	25.5	3.47	2.51–4.79	56.86 (1)	<0.0001	2.50	1.76–3.54	26.62 (1)	<0.0001	2.37	1.66–3.39	22.51 (1)	<0.0001
p for trend						78.66 (1)	<0.0001			32.50 (1)	<0.0001			27.53 (1)	<0.0001
Job strain	3,935	533	13.5												
Low strain	953	97	10.2	1.00				1.00				1.00			
Active	1,236	128	10.4	1.07	0.80–1.43	0.18 (1)	0.67	0.98	0.72–1.33	0.02 (1)	0.88	0.94	0.69–1.29	0.13 (1)	0.72
Passive	1,171	199	17.0	1.53	1.16–2.03	9.00 (1)	0.003	1.19	0.88–1.63	1.27 (1)	0.26	1.17	0.85–1.60	0.91 (1)	0.34
High strain	575	109	19.0	2.05	1.50–2.80	20.01 (1)	<0.0001	1.55	1.11–2.16	6.72 (1)	0.010	1.52	1.09–2.14	5.94 (1)	0.015
Number of close people	3,809	514	13.5												
7+	1,117	122	10.9	1.00				1.00				1.00			
3–6	1,919	275	14.3	1.33	1.05–1.68	5.74 (1)	0.017	1.19	0.94–1.52	2.03 (1)	0.15	1.26	0.98–1.62	3.27 (1)	0.07
0–2	773	117	15.1	1.49	1.13–1.97	7.97 (1)	0.005	1.40	1.04–1.88	5.04 (1)	0.025	1.51	1.12–2.03	7.13 (1)	0.008
p for trend						8.39 (1)	0.004			5.07 (1)	0.024			7.31 (1)	0.007

*Notes:* All analyses are based on multivariable logistic regression analysis. df: degrees of freedom.

a Adjusted for sex, length of follow-up time, and age at follow-up.

b Additionally adjusted for occupational position (except in analysis of occupational position), psychological distress at baseline and during follow-up, and marital status at follow-up.

c Additionally adjusted for long-standing illness, coronary heart disease, smoking, alcohol use, physical function, and income level at follow-up.

In Table 2 we consider associations between the overall number of midlife adversities and the number of adversities by category and symptoms of depression post-retirement. In addition to socioeconomic and psychosocial, we further grouped the exposures into work related and non-work related adversities. There was a clear trend in all groups suggesting that an increase in the number of adversities was associated with an increased risk of depressive symptoms. Mutual adjustment of socioeconomic adversity by psychosocial adversity and vice versa did not affect the estimates, and differences between the groups of adversities, based on overlapping confidence intervals, are unlikely to be significant.

**Table 2:** Odds Ratios (OR) and 95% Confidence Intervals (CI) Comparing Risk for Post-Retirement Depressive Symptoms by the Number and Type of Midlife Adversities

Number and Type of Midlife Adversities	Post-Retirement Depressive Symptoms										
	N	N of cases	%	OR^a^	95% CI^a^	Wald χ^2^ (df)	p	OR^b^	95% CI^b^	Wald χ^2^ (df)	p
Number of any adversities											
0	2,063	203	9.8	1.00				–	–		
1	1,379	212	15.4	1.34	1.05–1.69	5.71 (1)	0.017	–	–		
2–4	356	96	27.0	2.56	1.85–3.54	32.01 (1)	<0.0001	–	–		
p for trend						28.75 (1)	<0.0001	–	–		
Number of socioeconomic adversities (low occupational grade, poor standard of living)											
0	3,026	333	11.0	1.00				1.00			
1	736	165	22.4	1.70	1.31–2.22	15.36 (1)	<0.0001	1.71	1.31–2.23	15.54 (1)	<0.0001
2	36	13	36.1	3.20	1.41–7.25	7.79 (1)	0.004	3.34	1.48–7.58	8.35 (1)	0.004
p for trend						21.23 (1)	<0.0001			20.42 (1)	<0.0001
Number of psychosocial adversities (high job strain,^c^ low number of close relationships)											
0	2,607	323	12.4	1.00				1.00			
1	1,061	154	14.5	1.21	0.96–1.53	2.50 (1)	0.11	1.23	0.97–1.56	2.92 (1)	0.09
2	130	34	26.2	2.17	1.35–3.48	10.32 (1)	0.001	2.19	1.37–3.52	10.61 (1)	0.001
p for trend						9.44 (1)	0.002			10.20 (1)	0.001
Number of work related adversities (low occupational grade, high job strain^c^)											
0	2,768	296	10.7	1.00				1.00			
1	951	197	20.7	1.52	1.19–1.94	11.24 (1)	0.001	1.50	1.18–1.92	10.62 (1)	0.001
2	79	18	22.8	2.28	1.22–4.27	6.60 (1)	0.010	2.30	1.23–4.32	6.72 (1)	0.010
p for trend						14.70 (1)	0.0001			14.24 (1)	0.0002
Number of non-work related adversities (poor standard of living, low number of close relationships)											
0	2,846	353	12.4	1.00				1.00			
1	884	136	15.4	1.33	1.04–1.70	5.24 (1)	0.022	1.32	1.04–1.69	5.03 (1)	0.025
2	68	22	32.4	2.96	1.58–5.54	11.45 (1)	0.001	2.91	1.55–5.45	11.10 (1)	0.001
p for trend						12.87 (1)	0.0003			12.46 (1)	0.0004

*Notes:* All analyses are based on multivariable logistic regression analysis. df: degrees of freedom.

a Adjusted for sex, psychological distress at baseline and during follow-up, length of follow-up; age, marital status, longstanding illness, coronary heart disease, physical function, smoking, alcohol use, and income level at follow-up.

b Socioeconomic and psychosocial adversities additionally adjusted for each other; work related and non-work related adversities additionally adjusted for each other.

c No exposure group includes participants with low job strain, active, and passive job.

Table 3 shows that all covariates measured at follow-up were associated with symptoms of depression at follow-up: female sex, single, divorced/separated, or widowed marital status, low income, having longstanding illness or coronary heart disease, smoking, moderate alcohol use (showing lower odds for depression compared with no use or heavy use), and low physical function. The highest prevalence of depressive symptoms was found among widowed participants (25.4%) and those with low physical function (22.2%).

**Table 3:** Odds Ratios (OR) and 95% Confidence Intervals (CI) Comparing Risk for Post-Retirement Depressive Symptoms by Exposure to Post-Retirement Characteristics

Post-Retirement Characteristic	Post-Retirement Depressive Symptoms						
	N	N of cases	%	OR^a^	95% CI^a^	Wald χ^2^ (df)	p
All	3,939	534	13.6				
Sex							
Male	2,789	318	11.4	1.00			
Female	1,150	216	18.8	1.79	1.49–2.17	36.88 (1)	<0.0001
Marital status							
Married/ cohabiting	2,919	315	10.8	1.00			
Single	472	92	19.5	1.77	1.36–2.30	18.04 (1)	<0.0001
Divorced/separated	265	55	20.8	1.94	1.40–2.69	15.81 (1)	<0.0001
Widowed	283	72	25.4	2.75	2.02–3.74	41.10 (1)	<0.0001
Income level							
High	1,024	127	12.4	1.00			
Average	1,570	148	9.4	0.78	0.61–1.00	3.76 (1)	0.05
Low	1,345	259	19.3	1.65	1.30–2.08	17.42 (1)	<0.0001
Long-standing illness							
No	1,259	109	8.7	1.00			
Yes	2,680	425	15.9	2.08	1.66–2.60	40.68 (1)	<0.0001
Coronary heart disease							
No	3,339	428	12.8	1.00			
Yes	600	106	17.7	1.68	1.32–2.13	17.71 (1)	<0.0001
Smoking							
No	3,651	478	13.1	1.00			
Yes	288	56	19.4	1.58	1.16–2.15	8.33 (1)	0.004
Alcohol use							
No	704	148	21.0	1.00			
Moderate	2,954	336	11.4	0.54	0.43–0.68	29.26 (1)	<0.0001
Frequent/heavy	281	50	17.8	0.96	0.67–1.39	0.04 (1)	0.84
Physical function							
High	1,297	92	7.1	1.00			
Average	1,184	119	10.1	1.62	1.21–2.16	10.71 (1)	0.001
Low	1,458	323	22.2	4.22	3.26–5.47	118.29 (1)	<0.0001

*Notes:* All analyses are based on multivariable logistic regression analysis. df: degrees of freedom.

a Adjusted for age and sex.

Supplementary Table 1 (available online) shows the association between repeat exposure to psychological distress (at baseline and during the follow-up years) and symptoms of depression post-retirement. The odds ratio among participants having psychological distress measured at baseline was 2.62-fold, a result little affected by health-related factors and income level at follow-up. When participants with repeated psychological distress (i.e., distress both at baseline and at least once during follow-up) were compared with those with no such symptoms at either time, the odds ratio for CESD-depressive symptoms was 7.65. In this group the prevalence of depressive symptoms at follow-up was as high as 26.8%. The corresponding odds ratio appeared to be lower among participants with new onset psychological distress during follow-up (4.03) and for those with distress only at baseline (1.87). Adjustment for covariates had little effect on these associations.

## Discussion

Prospective data from nearly 4,000 men and women from the Whitehall II study suggest that socioeconomic and psychosocial adversity in midlife, whether work-related or non–work-related, is associated with symptoms of depression post-retirement, two decades later. These associations were only partly attributable to baseline mental health; onset of psychological symptoms during the follow-up years; or sociodemographic factors, physical health, and health behaviors after retirement.

Conventional prospective analyses assess the association between the exposure measured at one time point, the baseline, with the outcome measured at follow-up, with a robust association interpreted as evidence of risk factor status for the exposure. These studies, however, usually do not take account of bias due to confounding factors at follow-up (in this case, possible deterioration of physical health, health behaviors, and socioeconomic circumstances in retirement). In the present study we controlled the models for several important post-retirement factors and were able to reduce the risk of bias due to confounding and associated reverse causation. This is, for example, a situation in which adversity in midlife leads to post-retirement poor physical function which, in turn, is associated with post-retirement depression. In our study the adjustment of socioeconomic adversity for psychosocial adversity and vice versa ensured that the associations observed were not driven by one category of adversity.

Midlife, usually characterized by employment, has been viewed as a separate phase from post-retirement life. Although many changes occur at retirement, our data suggest that work exposures, like low occupational position and high job strain, may act as proxy measures for a wide range of unfavorable socioeconomic and psychosocial factors operating across the life course. Occupational position encapsulates many factors beyond the workplace, being related to educational and social background, status, self-esteem, income, and living conditions.^21^ In our study, position in the occupational hierarchy played a role in generating variation in the prevalence of depressive symptoms well after the burden of work had been lifted. Another socioeconomic predictor of post-retirement depressive symptoms was dissatisfaction with standard of living, a measure typically involving cumulative disadvantage across the adult life course.^3^

The association we found between psychosocial adversities in midlife, job strain and few close relationships, and symptoms of depression post-retirement is in line with previous studies showing a link between work stress, loneliness, living alone, and mental ill-health among middle-aged and elderly populations.^2^, ^3^, ^13^, ^14^, ^16^, ^17^, ^18^, ^32^, ^33^, ^34^, ^35^ Only one study, however, investigated the association between work stress before and SF-36 mental well-being after retirement^22^; thus, ours appear to be the first study in which these associations have been investigated in relation to a variety of midlife and old-age risk factors and post-retirement depressive symptoms.

Our findings can be interpreted within the life cycle framework of stress and depression, presented by Lupien and colleagues.^36^ According to that model, brain regions undergoing the most rapid age-related decline due to aging (hippocampus, frontal cortex, amygdala) are highly vulnerable to the effects of stress hormones. Unlike in childhood and adolescence when the brain is still developing and when programming (effects on the structure and function of brain tissues) may occur, adverse effects of stress exposure during adulthood can manifest itself as incubated effects of early adversity, or as maintenance of chronic effects of stress in adulthood. Early adversities might also make an individual more vulnerable to the effects of later exposures.

A limitation in the present study is the middle-aged cohort already at study entry; thus no prospective data on childhood adversities, such as socioeconomic disadvantage, abuse, and neglect, were available, therefore we cannot exclude the possibility that socioeconomic and psychosocial adversities measured in midlife in this study partly represent early life exposures.^37^ However, better understanding of the origins of post-retirement depressogenic effects can inform the most relevant life stages to be targeted by prevention.

Major strengths of this study are that it was based on a large data set and a follow-up of over two decades as well as control for several confounding factors at baseline and post-retirement. A limitation is that the CES-D was developed to identify individuals with depressive symptomatology; it is not designed to make a psychiatric diagnosis of major depressive disorder even if it has good criterion validity as a measure of depressive disorder, also in the Whitehall study.^31^

We assessed baseline mental health using the GHQ-30 questionnaire rather than a psychiatric interview or a clinical depression scale, although the GHQ is a well-established scale for the evaluation of psychological morbidity in general population samples. In relation to diagnosed mental disorders, especially depression, the GHQ has high clinical validity,^30^, ^38^ also confirmed in the Whitehall study cohort.^31^ As the GHQ also detects a range of minor psychiatric morbidities, such as subclinical depression, it is possible that our baseline adjustment using the GHQ is overzealous. We showed, however, that psychological distress at baseline and during follow-up years was strongly associated with CES-D depressive symptoms; the strongest predictor being repeated psychological distress. This is in agreement with an earlier report from the Whitehall study demonstrating that recurrent psychological distress is associated with a progressively increasing risk of future distress.^39^

As exposures and the outcome in our analysis were based on self-reports, these may introduce reporting bias—which is a problem, especially in studies of mental health^17^, ^40^, ^41^ (even in longitudinal studies in which baseline mental health has been controlled for). In our study, occupational position is unlikely to be severely affected by reporting bias because it is based on one's job title in the civil service. Nevertheless, future studies, ideally, should aim to find alternative, more objective ways to assess midlife exposures. We are aware that our measure of psychosocial adversity, which used job strain as proxy for such adversity in the workplace and number of close persons as a proxy outside of work, will not capture the full range of potential psychosocial adversities that are relevant in mid-life. This is a limitation of our study that we hope future studies will be in a position to rectify. Finally, the study population of white-collar civil servants limits generalizations of the findings, although there was a tenfold salary difference between the bottom and top grades in our study, suggesting that reduced variation in socioeconomic circumstances is an unlikely source of bias in the observed associations. As in all occupational cohorts, our sample is likely to be healthier and less exposed to adversities than the general population, potentially providing an underestimate of the association between the exposures and the outcome. Of the eligible participants (i.e., the total cohort excluding non-retired participants, N = 7,645), 38% were lost to follow-up. Previous analysis of the Whitehall II participants showed non-participation to be associated with increased risk of mortality.^42^ A major bias is unlikely, however, as there was no evidence that socioeconomic position modified the effect of non-response on mortality.

This 21-year follow-up of a cohort of middle-aged British individuals suggests that socioeconomic and psychosocial disadvantage in midlife—during employment—is associated with depressive symptoms in old age. These findings have practical implications as several socioeconomic and psychosocial risk factors for late-life depression can be detected in midlife and are potentially modifiable. Our results also suggest that observed associations are not explained by reverse causation or later-life socioeconomic adversity, poor physical health, or health-risk behaviors. Further research is needed to examine whether early identification of midlife socioeconomic and psychosocial risk factors and interventions aimed at reducing these risk factors will promote healthy aging and prevent depressive symptoms in old age.

## Figures and Tables

**Supplementary Table 1:** Odds Ratios (OR) and 95% Confidence Intervals (CI) Comparing Risk for Post-Retirement Depressive Symptoms by Psychological Distress at Baseline and During the Follow-up Years

Psychological Distress at Baseline and During the Follow-up Years	Post-Retirement Depression										
	N	N of cases	%	OR^a^	95% CI^a^	Wald χ^2^ (df)	p	OR^b^	95% CI^b^	Wald χ^2^ (df)	p
At baseline											
No	2,875	298	10.4	1.00				1.00			
Yes	1,064	236	22.2	2.62	2.14–3.21	86.74 (1)	<0.0001	2.46	2.00–3.03	71.92 (1)	<0.0001
At baseline and during follow-up											
No – No (healthy)	1,620	83	5.1	1.00				1.00			
No – Yes (onset)	1,255	215	17.1	4.03	3.04–5.36	92.87 (1)	<0.0001	3.49	2.62–4.66	72.09 (1)	<0.0001
Yes – No (improved)	272	24	8.8	1.87	1.14–3.07	6.05 (1)	0.014	1.83	1.11–3.03	5.53 (1)	0.019
Yes – Yes (repeated)	792	212	26.8	7.65	5.71–10.25	185.62 (1)	<0.0001	6.51	4.83–8.77	151.44 (1)	<0.0001

*Notes:* All analyses are based on multivariable logistic regression analysis. df: degrees of freedom.

a Adjusted for sex, occupational position (baseline), length of follow-up; age, and marital status at follow-up.

b Additionally adjusted for longstanding illness, coronary heart disease, physical function, smoking, alcohol use, and income level at follow-up.
